# A Note on the Effects of Linear Topology Preservation in Monte Carlo Simulations of Knotted Proteins

**DOI:** 10.3390/ijms232213871

**Published:** 2022-11-10

**Authors:** João N. C. Especial, Antonio Rey, Patrícia F. N. Faísca

**Affiliations:** 1 Departamento de Física, BioISI-Biosystems and Integrative Sciences Institute, Faculdade de Ciências, Universidade de Lisboa, 1749-016 Lisboa, Portugal; 2 Departamento de Química Física, Facultad de Ciencias Químicas, Universidad Complutense, 28040 Madrid, Spain

**Keywords:** knotted proteins, protein folding, off-lattice model, Monte Carlo simulations

## Abstract

Monte Carlo simulations are a powerful technique and are widely used in different fields. When applied to complex molecular systems with long chains, such as those in synthetic polymers and proteins, they have the advantage of providing a fast and computationally efficient way to sample equilibrium ensembles and calculate thermodynamic and structural properties under desired conditions. Conformational Monte Carlo techniques employ a move set to perform the transitions in the simulation Markov chain. While accepted conformations must preserve the sequential bonding of the protein chain model and excluded volume among its units, the moves themselves may take the chain across itself. We call this a break in linear topology preservation. In this manuscript, we show, using simple protein models, that there is no difference in equilibrium properties calculated with a move set that preserves linear topology and one that does not. However, for complex structures, such as those of deeply knotted proteins, the preservation of linear topology provides correct equilibrium results but only after long relaxation. In any case, to analyze folding pathways, knotting mechanisms and folding kinetics, the preservation of linear topology may be an unavoidable requirement.

## 1. Introduction

Knotted proteins are globular proteins whose native structure embeds a physical (i.e., open) knot. The first mention of knotted proteins in the literature dates back to 1977 [1], but these tangled macromolecules did not attract much attention until 2000 when Taylor developed a knot detection method, the so-called Koniaris–Muthukumar–Taylor (KMT) algorithm, which is able to determine if an open polypeptide chain is knotted [2]. The use of loop closure procedures, combined with knot detection and identification methods [3], allowed the Protein Data Bank (PDB) [4] to be probed for knotted proteins. According to the latest survey, the PDB contains about 1% knotted proteins [5]. The most frequent knot type found in proteins is the $3_{1}$ (or trefoil knot), with three crossings on a planar projection, and the most complex protein knot found in the PDB is the $6_{1}$ (or Stevedore’s) knot [6], with six crossings on a planar projection. Interestingly, a $7_{1}$ knot was recently detected in the AlphaFold database [7]. Knots in proteins can be further classified as shallow or deep, according to the location of the knotted core [8], the minimal segment of the polypeptide chain that contains the knot. If the latter is located only a few residues (say, less than 15) away from one of the protein termini, the corresponding knot tail is said to be short and the knot classified as shallow; otherwise, the knot tail is long, and the knot is considered deep.

Over the last 15 years, researchers have dedicated considerable attention to knotted proteins. Determining their functional role (if any) [9] and unravelling their folding and knotting mechanisms are examples of fundamental questions that have been extensively explored through experiments in vitro and via molecular simulations (reviewed in [10,11,12]). The effects of knot deepness have also been investigated [13] as deep knots are expected to complicate the folding process. Monte Carlo (MC) methods and Molecular Dynamics simulations, often combined with parallel tempering (or replica-exchange) schemes [14,15], have been used to sample the conformational space of knotted model systems spanning different levels of resolution, from simple cubic lattices [16,17,18] and C-alpha models [19,20,21,22,23,24] to full atomistic representations [25,26,27]. Due to its lower computational cost, replica-exchange MC (RE-MC) is perhaps the most efficient method to sample canonically distributed conformational states across different temperatures and evaluate equilibrium properties [15]. An example of the latter is the melting temperature, $T_{m}$ (the temperature at which the heat capacity peaks), which provides information on thermal stability—the higher the $T_{m}$, the more thermally stable the protein.

In a typical MC folding simulation, a Markov chain of random conformations is generated. In particular, at each MC step, a move randomly selected from a move set generates a random trial conformation, which preserves the covalent nature of the chain and accounts for the excluded volume. Given the polymeric nature of the polypeptide chain, the move set usually comprises movements originally designed for polymer physics simulations [28]. This means that when conducting off-lattice simulations to evaluate equilibrium properties, moves that take the backbone across itself are considered valid, as long as the final conformation is free of steric clashes. In the test tube and in the cell, however, the conformational drift that takes the protein from the (more or less) extended conformation from which it is released from the ribosome to its native structure does not allow the backbone to cross itself due to the excluded volume. Consequently, the folding process necessarily preserves the linear topology of the polypeptide chain. It would thus be natural to expect that a simulation of protein folding should also enforce this kind of topology preservation. This would certainly be compulsory if one is interested in evaluating kinetic properties, determining the folding pathway or the knotting mechanism. However, as long as the chosen move set is ergodic, it cannot affect equilibrium properties, and since the preservation (or not) of the linear topology is a property of the move set, it cannot affect these properties either.

In the present work, we investigate the consequences of linear topology preservation in off-lattice MC simulations of simple C-alpha models of knotted and unknotted proteins. We find, as expected, that equilibrium properties of knotted and unknotted proteins are not influenced by enforcing linear topology preservation. Nevertheless, in the case of deeply knotted proteins, we find that linear topology preservation considerably hinders relaxation to thermal equilibrium, with topology preserving simulations requiring up to two orders of magnitude more Monte Carlo steps to equilibrate than non-topology preserving simulations of the same model system. In addition, we find that for knotted proteins, thermal equilibrium properties are mostly determined by the structure of the knotted core and independent of the length of the knot tails, at least when these are unstructured.

## 2. Results

### 2.1. Model Systems

**Figure 1:**
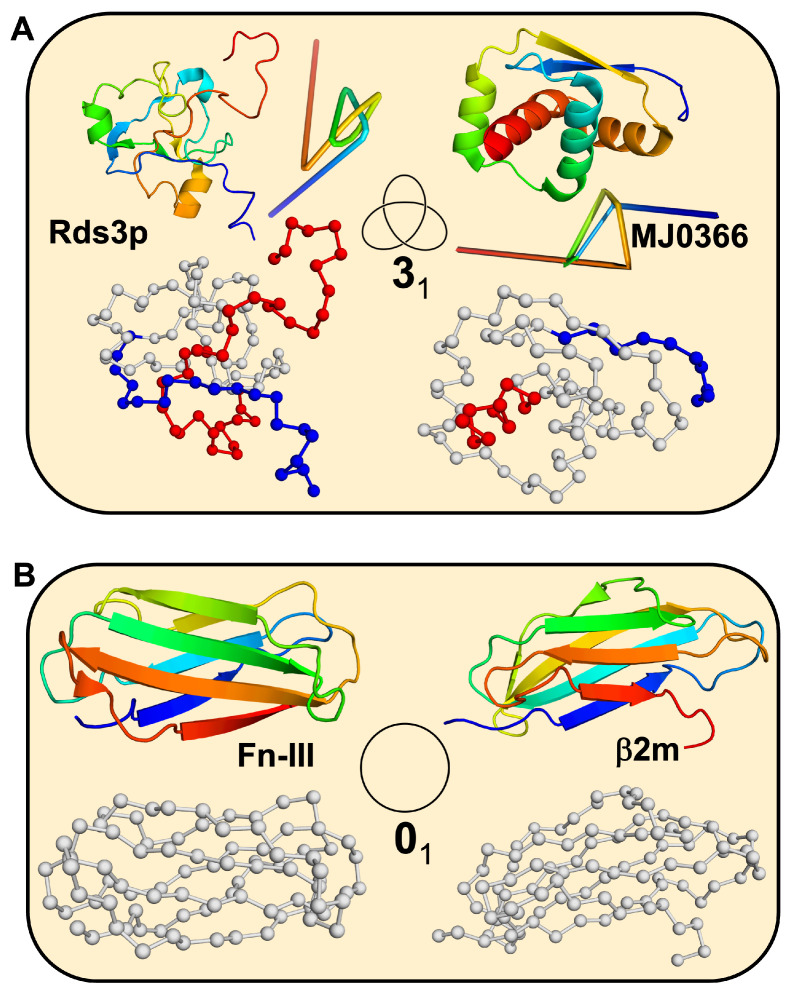
Model systems. (**A**) Knotted proteins: Rds3p (PDB id: 2K0A), MJ0366 (PDB id: 2EFV); residues in the N-tail are colored blue, residues in the knotted core are colored grey, and residues in the C-tail are colored red; reduced representations of the knots obtained from the protein knots server [35]. (**B**) Unknotted proteins: Fn-III (PDB id: 1TEN), β2m (PDB id: 1BMG).

In this work, we focus on two knotted proteins (Figure 1A). The first one, Rds3p (PDB id: 2K0A [29]), is 109 residues long and embeds a trefoil knot. The knotted core extends from residue 21 to 74. The N-tail of the knot is 20 residues long, and the C-tail comprises 35 residues. Therefore, the knot is classified as deep. The second knotted protein, MJ0366 (PDB id: 2EFV [30]), is the smallest (92 residues long) knotted protein found to date. Its native structure also embeds a trefoil knot. In this case, the knotted core comprises residues 11 to 82. Since both knot tails are short (10 residues), the knot is classified as shallow.

We also analyzed two beta-sandwich proteins that are unknotted (Figure 1B): the fibronectin type domain (Fn-III) from tenascin (PDB id: 1TEN [31]) and bovine beta-2-microglobulin (β2m) (PDB id: 1BMG [32]). Their sizes (90 and 98 residues, respectively) are similar to those of the knotted proteins we are considering. We have chosen these beta proteins because they also have two-state folding transitions, and, in general terms, beta proteins show more cooperative transitions than alpha proteins [33,34].

### 2.2. LTyP and Equilibrium Sampling

We started by verifying that linear topology preservation (LTyP) does not influence equilibrium sampling for the knotted and unknotted proteins considered in this study. In an MC folding simulation, for a given protein, the same final equilibrium distribution must be achieved no matter the region of conformational space from which the simulation starts and no matter what move set is used—LTyP or non-LTyP. We have thus conducted sets of four RE-MC simulations. In two of them, all replicas at different temperatures start from the same denatured conformation, while in the other two, the replicas start from the native structure. In each of these pairs, one of the simulations was LTyP and the other non-LTyP. Our idea is that if thermal equilibrium is achieved at every temperature of the RE ladder, the results of these four simulations cannot differ and, therefore, comparisons among the simulation results can be a test for proper equilibration.

**Figure 2:**
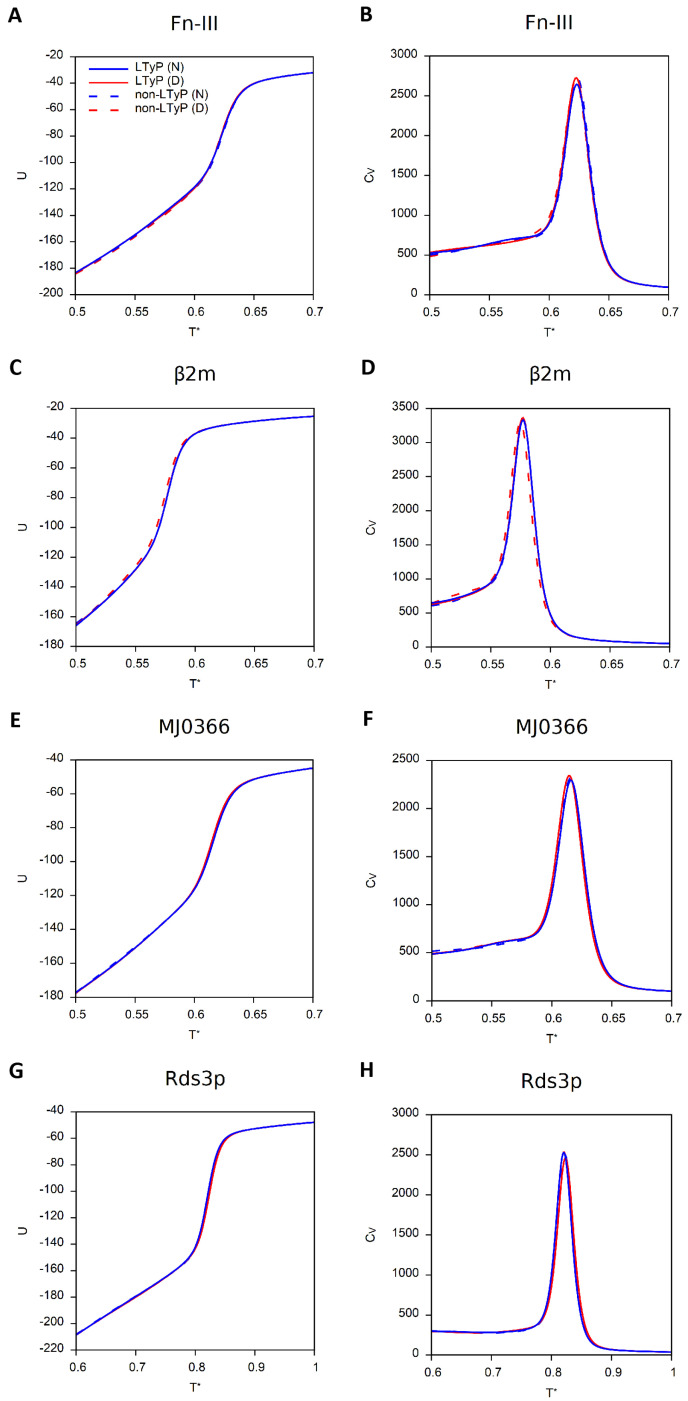
Internal energy, *U*, and heat capacity, $C_{V}$, as functions of the reduced temperature, $T^{*}$ (see Section 4). Simulations starting from the native conformation (N) are colored blue, and those starting from a denatured conformation (D) are colored red. Continuous lines correspond to LTyP simulations and dashed lines to non-LTyP simulations. (**A**,**B**) Fn-III (PDB id: 1TEN). (**C**,**D**) β2m (PDB id: 1BMG). (**E**,**F**) MJ0366 (PDB id: 2EFV). (**G**,**H**) Rds3p (PDB id: 2K0A).

In Figure 2, we report the dependence on *T* of the internal energy, *U* (computed from the energies of the conformations sampled at a given temperature in accordance with the interaction potential of Equation (4) in Section 4.1) and $C_{V}$, for the four model systems considered in this study.

**Figure 3:**
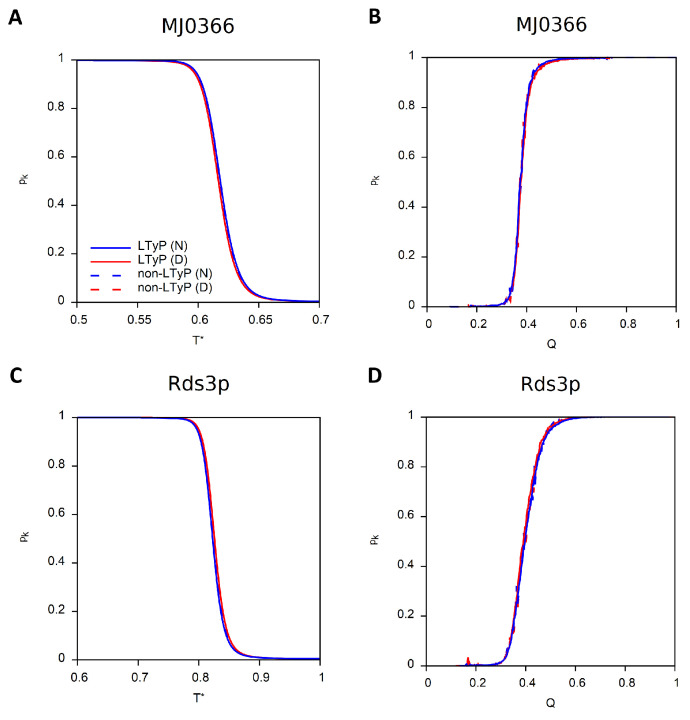
Knotting probability, $p_{k}$, as function of reduced temperature and of *Q*. Simulations starting from native conformation (N) are colored blue and those starting from a denatured conformation (D) are colored red. Continuous lines correspond to LTyP simulations and dashed lines to non-LTyP simulations. (**A**,**B**) MJ0366 (PDB id: 2EFV). (**C**,**D**) Rds3p (PDB id: 2K0A).

For the two knotted proteins, we also present the dependence of the knotting probability ($p_{k}$) on *T* as well as on the reaction coordinate *Q*, defined as the fraction of native contacts formed (Figure 3). At the lowest temperature, the conformational ensemble is expected to be fully populated by the knotted native structure and, thus, the knotting probability should tend to 1 at low *T*. Similarly, the probability of finding knotted conformations is expected to achieve its maximum value in ensembles of conformations that are native or nearly native (i.e., having high *Q*).

The equilibrium results of the four simulations performed for each model system coincide, as expected, but the number of mcs (Monte Carlo steps) required for each simulation to relax to thermal equilibrium may differ by orders of magnitude, as reported in Table 1.

When simulations preserve linear topology, knotted proteins require considerably more mcs to equilibrate, particularly when the simulation starts from a denatured conformation, and even more so when the knot is deep.

**Figure 4:**
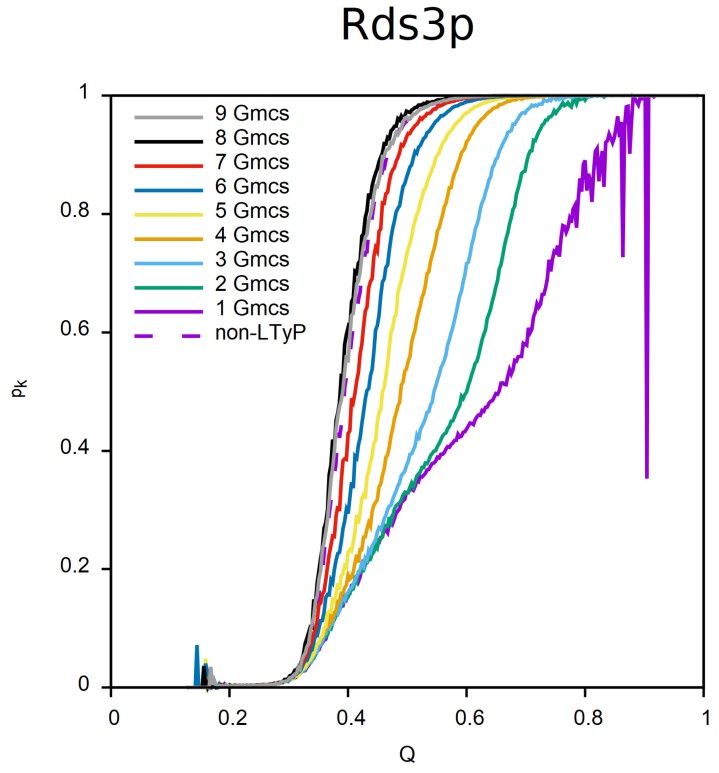
Progress of relaxation for LTyP simulations of protein Rds3p starting from a denatured conformation. Knotting probability, $p_{k}$, as function of the reaction coordinate, *Q*, for numbers of relaxation mcs ranging from $10^{9}$ (1 Gmcs) to $9\times 10^{9}$ (9 Gmcs).

To illustrate the progress of the relaxation process under LTyP, we report in Figure 4 the knotting probability, $p_{k}$, as function of *Q*, for protein Rds3p and simulations starting from a denatured conformation, for numbers of mcs of relaxation ranging from $10^{9}$ (1 Gmcs) to $9\times 10^{9}$ (9 Gmcs). For this particular case, only after $8\times 10^{9}$ mcs can the simulation be considered in equilibrium.

### 2.3. LTyP and Deep Knots

**Figure 5:**
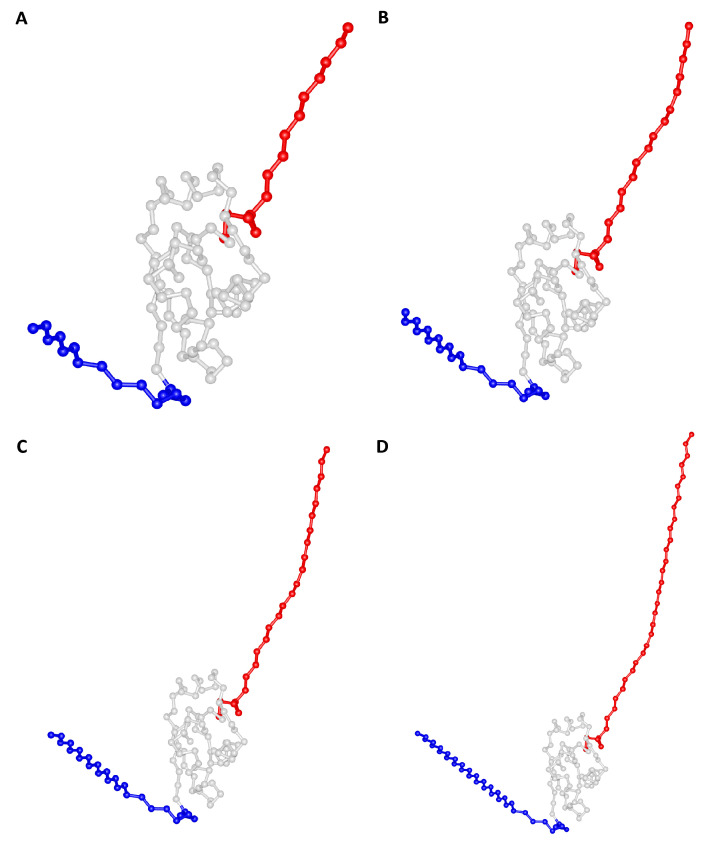
MJ0366 with (**A**) 5 alanines added to each tail resulting in N and C tails with 15 residues (N15C15), (**B**) 10 alanines added to each tail resulting in N and C tails with 20 residues (N20C20), (**C**) 15 alanines added to each tail resulting in N and C tails with 25 residues (N25C25) and (**D**) 25 alanines added to each tail resulting in N and C tails with 35 residues (N35C35); residues in the N-tail are colored blue, residues in the knotted core are colored grey, and residues in the C-tail are colored red.

Should LTyP influence the number of mcs required for simulations of deeply knotted proteins to equilibrate, then, by increasing the size of the knot tails of protein MJ0366 and keeping the number of relaxation mcs fixed, one should, at some point, begin to obtain different results for LTyP simulations starting from native and from denatured conformations, indicating that the simulation was no longer able to reach equilibrium. Furthermore, the deviation in the results should become larger as the size of the knot tails increases and, for tails of comparable size, become similar to that observed for Rds3p for similar number of mcs of relaxation. To test this hypothesis, we used PyMol to prepare four engineered variants of MJ0366 to which 5 (Figure 5A), 10 (Figure 5B), 15 (Figure 5C) and 25 (Figure 5D) alanine residues were added to both termini in an approximate straight line to minimize the addition of spurious native contacts. Our aim is just to introduce a topological burden to the relaxation, which, in turn, may result in a more difficult knotting step, without significantly modifying the energetic stability of the folded state.

**Figure 6:**
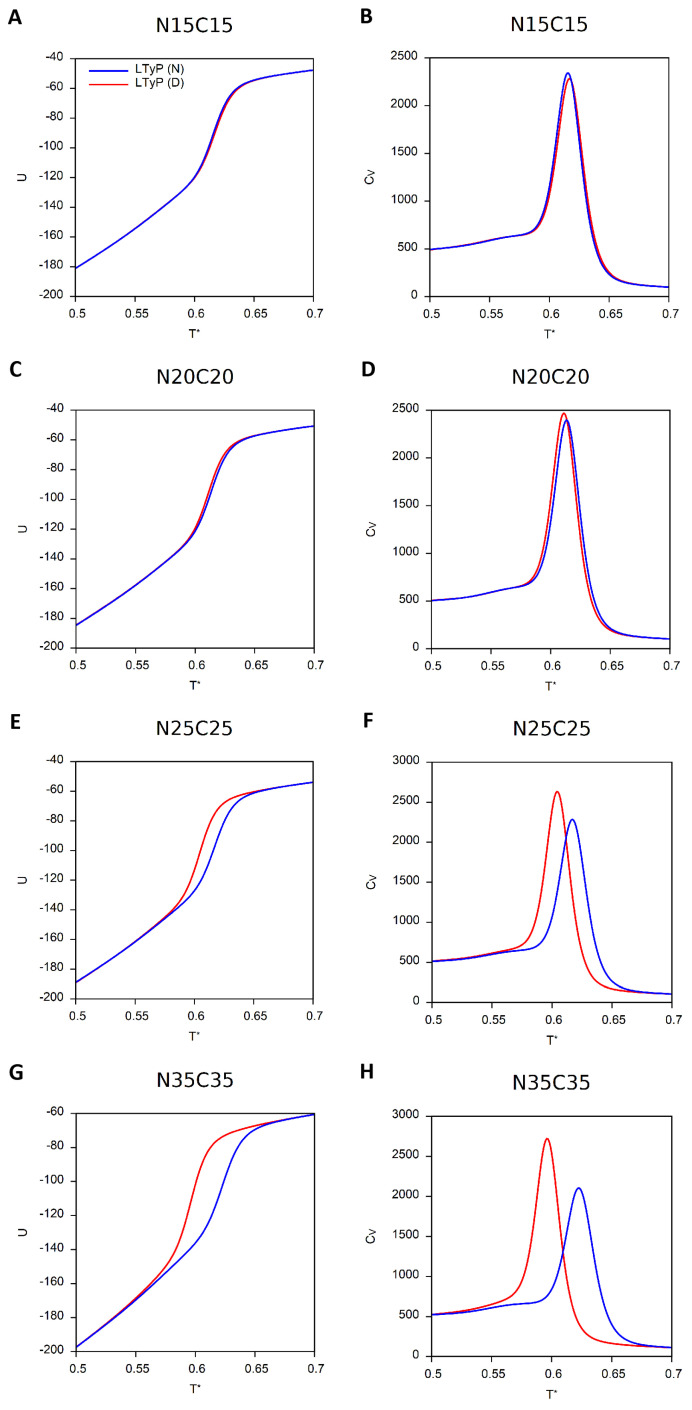
Internal energy, *U*, and heat capacity, $C_{V}$, as functions of reduced temperature for MJ0366 with tails of increasing length. Simulations starting from native conformation (N) are colored blue and those starting from a denatured conformation (D) are colored red. (**A**,**B**) N15C15. (**C**,**D**) N20C20. (**E**,**F**) N25C25. (**G**,**H**) N35C35.

**Figure 7:**
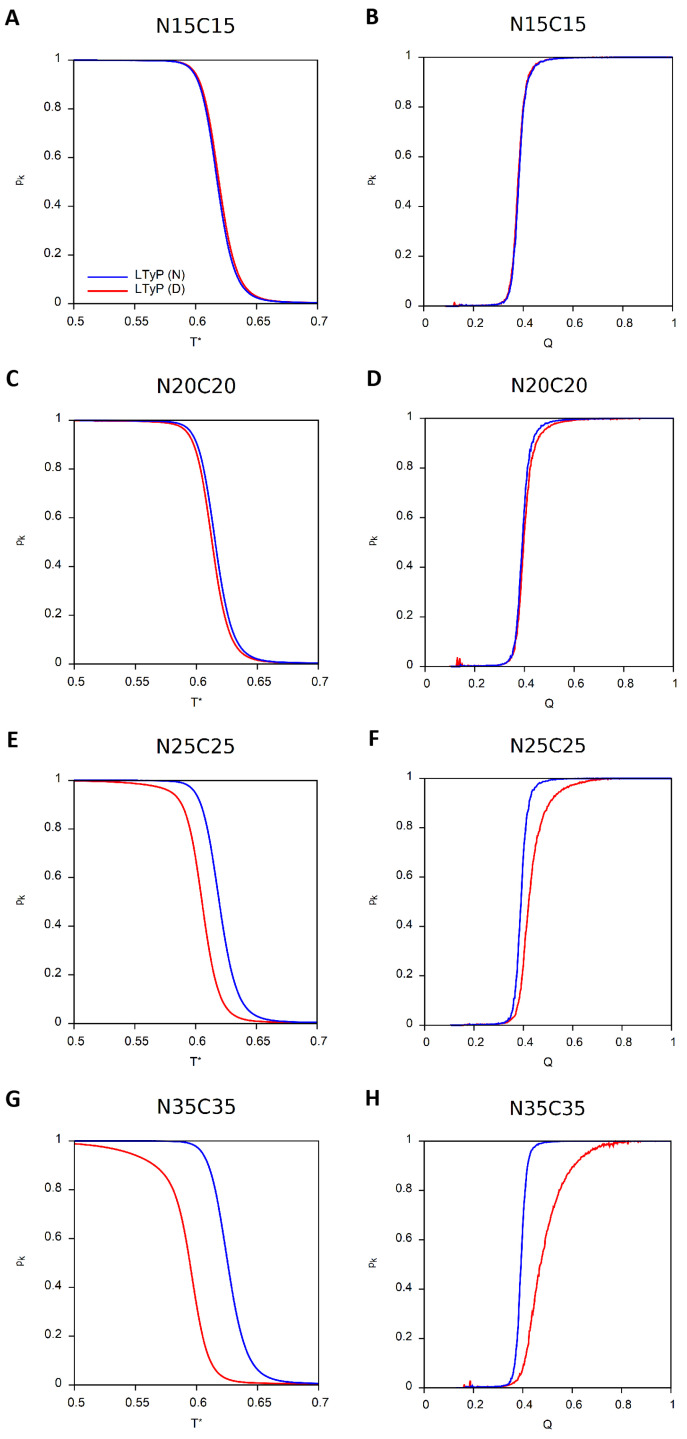
Knotting probability, $p_{k}$, as function of reduced temperature and of *Q* for MJ0366 with tails of increasing length. Simulations starting from native conformation (N) are colored blue and those starting from a denatured conformation (D) are colored red. (**A**,**B**) N15C15. (**C**,**D**) N20C20. (**E**,**F**) N25C25. (**G**,**H**) N35C35.

The conformations thus obtained were subsequently subjected to a MC simulation above $T_{m}$ to generate denatured (and unknotted) conformations to be used as starting conformations in the folding simulations. The results obtained in RE-MC simulations with a total of $10^{9}$ mcs ($5\times 10^{8}$ of which were used for relaxation) are reported in Figure 6A–H and Figure 7A–H and clearly support our hypothesis. When the tail length increases, the LTyP simulations starting from unfolded conformations deviate from those starting from the native conformation, indicating that the former no longer reached equilibrium in the considered number of mcs.

It is also interesting to note that the results of the simulations that start from the native structure, which are in equilibrium, show very little difference among them as tail lengths increase, indicating that for model systems in which the tails do not contribute significantly to the energetic stability of the native state, equilibrium properties are mostly determined by the structure of the knotted core.

## 3. Discussion

The study of protein folding encompasses the determination of the folding kinetics and mechanism and evaluating equilibrium properties, such as the melting temperature or free energy landscapes (i.e., projections of the free energy on one or more reaction coordinates). If the native state embeds a knot, determining the knotting mechanism is an additional challenge.

In nature, proteins explore their conformational space while preserving the linear topology of the polypeptide chain because the chain is not allowed to cross itself as it transitions between conformations. Therefore, it is natural to expect that a simulation protocol designed to study protein folding should mimic the natural process by enforcing the preservation of linear topology. This is perhaps even more true in the case of knotted proteins given the tangled nature of their native structure. In the case of Monte Carlo simulations, the preservation of the linear topology of the chain is determined by the move set used.

Equilibrium properties at a certain temperature, however, are exclusively determined by the energy through the potential function. Therefore, all simulations of a model system using the same potential must produce identical equilibrium results regardless of the initial conformation or of the move set used, if the latter is ergodic. This has been confirmed by the results reported here, which also highlight the need to verify that simulations starting from different regions of conformational space lead to the same results in order to ensure that thermal equilibrium has been reached. Given that replica-exchange Monte Carlo simulations aim to calculate equilibrium properties, a move set that does not preserve the linear topology is indeed recommended because it leads to thermal equilibrium in far fewer Monte Carlo steps, at least for proteins with complex native structures such as those with deep knots.

The results from present simulations show that increasing the knot depth by enlarging the knot tails has no effect on the melting temperature and, consequently, on the carrier protein’s thermal stability. Thus, proteins with deep knots are not expected to be more thermally stable than proteins with shallow knots, provided the knot tails are unstructured. In other words, if there is a functional role for deep knots in proteins, it should not be associated with equilibrium properties such as the melting temperature. This is in line with previous results reported in the scope of lattice models [13], which also indicated that the presence of a knot (shallow or deep) in the native structure is not a source of thermal stability. Indeed, the same melting temperature was obtained for a lattice knotted protein as well as for its unknotted counterpart.

Simulations that do not preserve the linear topology of the chain can be used to inform about equilibrium intermediate states but should not be adequate to establish the folding pathway or the knotting mechanism or to determine the folding rate.

## 4. Materials and Methods

### 4.1. Off-Lattice Model

The protein conformation is represented by a C-alpha model in which amino acids are reduced to hard spherical beads of uniform size, centered on the C-alpha atoms, and the covalent bonds that connect consecutive C-alpha atoms in the backbone are represented by rigid sticks, with the beads constituting joints with spherical degrees of freedom between these sticks. For the beads, we adopt a radius of 1.7 Å, which is the van der Waals radius of C-alpha atoms [36], and for the length of each stick, we adopt the distance between the C-alpha atoms of the respective bonded residues in the protein’s native conformation, varying from 2.9 Å (for cis bonds) to 3.8–3.9 Å (for trans bonds).

Two non-bonded residues are considered to be in contact in the native conformation if the smallest distance between any two heavy atoms, one belonging to each residue, is ≤4.5 Å; this cut-off is chosen because it is slightly larger than twice the average van der Waals radius of heavy atoms in proteins.

The total energy of a conformation defined by bead coordinates $\left\{{\vec{r}}_{i}\right\}$ is given by

(1) $$E\left(\{{\vec{r}}_{i}\}\right)=\varepsilon \sum_{i,j\geq i+2}^{N}\varphi \left(\frac{\left|{\vec{r}}_{i}-{\vec{r}}_{j}\right|-\left|{\vec{r}}_{i}^{\;nat}-{\vec{r}}_{j}^{\;nat}\right|}{w}\right)\left(\chi_{ij}\chi_{ij}^{nat}+\chi_{ji}\chi_{ji}^{nat}+\frac{1}{2}\right)\Delta_{ij}^{nat}\;,$$

where $\Delta_{ij}^{nat}$, the native contact map matrix (which takes the value 1 if the i−j contact is present in the native conformation and is 0 otherwise), ensures that only native contacts contribute to the energy, ε is a uniform intramolecular energy parameter (taken as −1 in this study, in which energies and temperatures are shown in reduced units), *N* is the chain length measured in number of beads, φ is the potential well associated with the native contacts, *w* is the half-width of this potential well, and the chirality of contact i−j in the conformation under consideration is

(2) $$\chi_{ij}=\Theta \left(\;{\left({\vec{r}}_{i}-{\vec{r}}_{j}\right)}^{}\cdot \left[\left({\vec{r}}_{j+1}-{\vec{r}}_{j}\right)\times \left({\vec{r}}_{j-1}-{\vec{r}}_{j}\right)\right]\;\right)-\frac{1}{2}\;.$$

The chirality of the i−j contact in the native conformation is

(3) $$\chi_{ij}^{nat}=\Theta \left(\;{\left({\vec{r}}_{i}^{\;nat}-{\vec{r}}_{j}^{\;nat}\right)}^{}\cdot \left[\left({\vec{r}}_{j+1}^{\;nat}-{\vec{r}}_{j}^{\;nat}\right)\times \left({\vec{r}}_{j-1}^{\;nat}-{\vec{r}}_{j}^{\;nat}\right)\right]\;\right)-\frac{1}{2}\;.$$

In Equations (2) and (3), Θ is Heaviside’s unit step function, which takes the value 1 if its argument is greater than zero and the value 0 otherwise. The chirality factor in (1) favors the native conformation *vis a vis* its mirror conformation, thereby ensuring chirality coherence among all contacts and the convergence of the simulations towards the native ensemble for temperatures below transition temperature.

In this study an inverse quadratic potential well is used. In this case, Equation (1) becomes

(4) $$E\left(\{{\vec{r}}_{i}\}\right)=\varepsilon \sum_{i,j\geq i+2}^{N}{\left[{\left(\frac{\left|{\vec{r}}_{i}-{\vec{r}}_{j}\right|-\left|{\vec{r}}_{i}^{\;nat}-{\vec{r}}_{j}^{\;nat}\right|}{w}\right)}^{2}+1\right]}^{-1}\left(\chi_{ij}\chi_{ij}^{nat}+\chi_{ji}\chi_{ji}^{nat}+\frac{1}{2}\right)\Delta_{ij}^{nat}\;.$$

The half-width of the potential well, *w*, determines the degree of cooperativity of the folding transition, with a wider well leading to less cooperative transitions occurring at higher transition temperatures. We measure the degree of cooperativity of the transition by the ratio of the full width at half maximum (FWHM) of the $C_{V}$ peak to the temperature at which the peak occurs—the melting temperature, $T_{m}$. A typical two-state transition that has been well characterized experimentally is that of the B1 domain of protein G (PDB id: 2GB1 [37]), and its $FWHM/T_{m}$ ratio at pH 5.4 has been determined to be approximately 4.4% [38]. Hence, the half-width of the potential well is adjusted to obtain a simulated $FWHM/T_{m}$ ratio between 4 and 5%. This is not just a technical detail of the model. As a matter of fact, very narrow transitions, which lead to artificially large cooperativity, can be poorly sampled with a replica exchange procedure such as the one used here [19,39,40]. The width proposed above has been successfully used in previous simulations employing a similar potential and sampling [19,20,41,42].

To identify which native contacts are formed in a sampled conformation, we consider that a native contact is formed if the distance between the centers of the respective beads differs from the distance between their C-alpha atoms in the native conformation by less than the half-width of the potential wells, *w*.

### 4.2. Monte Carlo Sampling

**Figure 8:**
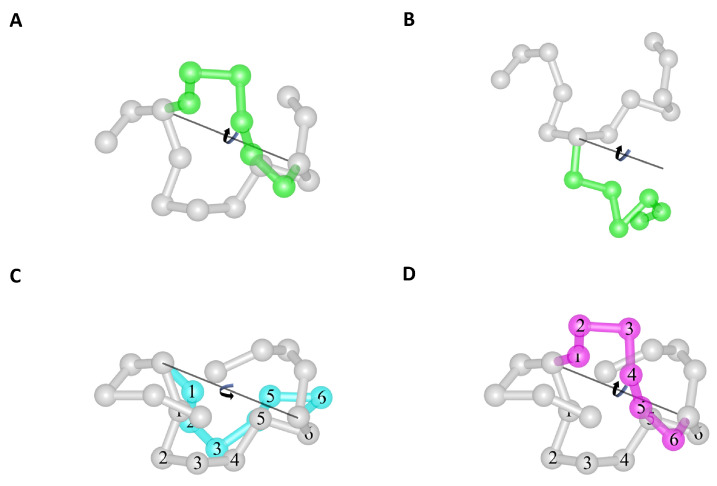
The move set. (**A**) Crankshaft move: Axis passes through two randomly selected beads and the beads between these are rotated around the axis. (**B**) Pivot move: Axis passes through one randomly selected bead, and the beads between that and a randomly selected terminus are rotated around the axis. (**C**) LTyP move variant: The rotated segment can reach the cyan conformation without passing across any fixed segment, hence the linear topology of the chain is preserved. (**D**) non-LTyP move variant: The magenta conformation can only be reached by the rotated segment by either moving across the front or across the back fixed segments; hence, the linear topology of the chain is not preserved in this crankshaft move.

To sample canonically distributed conformational states, we use Metropolis [43] MC-RE. The conformational space is explored using a move set that comprises two elementary moves: crankshaft (Figure 8A) and pivot (Figure 8B).

These elementary moves can be performed in either of two ways: (1) by limiting the amplitude of the rotation so that no bead or stick is allowed to overlap or move across another (e.g., Figure 8C); (2) by not limiting the rotation and allowing such overlaps and crossings to potentially occur (e.g., Figure 8D). A simulation that only performs moves of the first kind preserves the linear topology of the chain and is designated as a linear topology preserving (LTyP) simulation. Conversely, a simulation that allows moves that take the chain across itself is designated as non-LTyP.

To establish the clockwise and counter-clockwise rotation limits of LTyP moves, we note that both elementary moves only involve the rotation around an axis of a set of linked beads, which is performed in the vicinity of fixed beads that are also linked. For each pair (moving bead, fixed bead), we determine whether the moving bead may collide with the fixed bead and, if so, calculate the clockwise and counter-clockwise rotation angles that would cause the moving bead to come into contact without overlap with the fixed bead. These two angles define the free rotation interval of the moving bead relative to the fixed bead considered. The free rotation interval for the whole set of moving beads is the intersection of the free intervals of all (moving bead, fixed bead) pairs. Given that the maximum length of all bead links is 3.9 Å, if the radius of the beads is greater than or equal to 1.38 Å >(3.9 Å$/2)\left(\sqrt{2}/2\right)$, no pair of linked beads can be moved across another pair of linked beads without at least two beads overlapping. Hence, because all beads are linked and have a 1.7 Å radius, we ensure that no such crossing can occur, and linear topology is preserved. Whereas LTyP moves always generate trial conformations that are free of steric clashes, non-LTyP moves may produce trial conformations having steric clashes, and in non-LTyP simulations, such conformations are identified and rejected.

In a Monte Carlo step (mcs), a move is randomly selected from the two elementary possibilities mentioned above, with a probability of 0.5 for each. The number of beads moved is also randomly selected between 1 and the largest integer smaller than 3N/4, using a uniform distribution over this integer range. The fraction of native contacts, Q, is used as reaction coordinate, i.e., as an indicator of folding progress, with Q≈0 representing the denatured state and Q≈1 representing the native state. The topological state of a sampled conformation (i.e., knotted or unknotted) was determined using the KMT algorithm [2].

Simulations were initiated both from an unfolded conformation and from the native conformation. Simulations terminate after between $10^{8}$ and $10^{10}$ mcs. The first part of the simulation is used to allow all replicas to relax to the equilibrium ensemble at their particular temperature. For short simulations, those involving less than $2\times 10^{9}$ mcs, the initial half is assigned to equilibration. For longer simulations, those involving $n\times 10^{9}$ mcs with n≥2, $\left(n-1\right)\times 10^{9}$ mcs are assigned to equilibration. Relevant properties (E, Q, topological state, etc.) are recorded during the final part of the simulation, whether that be the final half for short simulations or the final $10^{9}$ mcs for longer ones. Sample elements are collected at every $10^{4}$ mcs. A temperature grid involving 64 distinct temperatures is used. These are non-uniformly distributed, the grid being denser in the vicinity of $T_{m}$ to ensure ample overlap of the canonical energy distributions of all temperature adjacent replicas, as required by RE. Replica exchange is attempted every $10^{3}$ mcs. Each replica is assigned a unique token, and replicas also exchange this token whenever conformations are exchanged. Tracing the evolution of the tokens throughout the simulation enabled the confirmation that RE was being performed adequately and that tokens underwent several round trips along the full temperature ladder during the simulations.

The Weighted Histogram Analysis Method (WHAM) [44] was used to analyze the sampled data and produce maximum likelihood estimates of the density of states, from which expected values for thermodynamic properties were calculated as functions of temperature. In particular, heat capacity, $C_{V}$, defined in reduced units as $C_{V}=\left(<E^{2}>-<E{>}^{2}\right)/T^{2}$, was evaluated as a function of temperature. The melting temperature, $T_{m}$, was determined as the temperature at which $C_{V}$ reached its maximum value, and the width of this peak at half-maximum was determined to calculate the $FWHM/T_{m}$ ratio. WHAM was also used to project the density of states along the chosen reaction coordinate to obtain knotting probability profiles along this coordinate.

## Figures and Tables

**Table 1:** Number of Monte Carlo steps (mcs) required for a simulation to achieve thermal equilibrium. Simulations starting from a denatured conformation are designated (D), and those starting from the native conformation are designated (N).

Protein	PDB Id	Non-LTyP (D)	Non-LTyP (N)	LTyP (D)	LTyP (N)
Fn-III	1TEN	$10^{8}$	$10^{8}$	$10^{8}$	$10^{8}$
β2m	1BMG	$10^{8}$	$10^{8}$	$10^{8}$	$10^{8}$
MJ0366	2EFV	$10^{8}$	$10^{8}$	$10^{9}$	$10^{8}$
Rds3p	2K0A	$10^{8}$	$10^{8}$	$10^{10}$	$10^{9}$

## Data Availability

Not applicable.
